# Mesangiogenic progenitor cells: a mesengenic and vasculogenic branch of hemopoiesis? A story of neglected plasticity

**DOI:** 10.3389/fcell.2025.1513440

**Published:** 2025-03-24

**Authors:** Simone Pacini

**Affiliations:** Department of Clinical and Experimental Medicine, University of Pisa, Pisa, Italy

**Keywords:** mesangiogenic progenitor cells, bone marrow cell culture, monocytes, macrophages, de-differentiation, trans-differentiation, OCT-4, nanog

## Abstract

Mesangiogenic progenitor cells (MPCs) are mesengenic and vasculogenic cells isolated from human bone marrow mononuclear cell cultures. Although MPCs were first described over two decades ago and have demonstrated promising differentiation capabilities, these cells did not attract sufficient attention from experts in the field of tissue regeneration. Several reports from the first decade of the 2000s showed MPC-like cells co-isolated in primary mesenchymal stromal cell (MSC) cultures, applying human serum. However, in most cases, these rounded and firmly attached cells were described as “contaminating” cells of hemopoietic origin. Indeed, MPC morphology, phenotype, and functional features evoke but do not completely overlap with those of cultured peripheral macrophages, and their hemopoietic origin should not be excluded. The plasticity of cells from the monocyte lineage is surprising but not completely unprecedented. Underestimated data demonstrated that circulating monocyte/macrophages could acquire broader plasticity under specific and different culture conditions, and this plasticity could be a consequence of *in vitro* de-differentiation. The evidence discussed here suggests that MPCs could represent the cell identity toward which the de-differentiation process reprograms the circulating mature phagocytic compartment.

## 1 Mesangiogenic progenitor cells (MPCs)

Mesangiogenic progenitor cells (MPCs) were first described in 2008 in cultures of human bone marrow mononuclear cells (hBM-MNC) aimed at isolating mesenchymal stromal cells (MSCs) under animal-free conditions (Petrini et al., 2009). In this work, we replaced fetal bovine serum (FBS), usually applied for the isolation/expansion of MSCs, with human autologous serum (hAS). This substitution apparently did not affect hBM-MSC culture; however, compared to standard FBS-supplemented cultures, the application of hAS led to the co-isolation of rounded, highly refringent, and firmly attached cells, with a frequency ranging from 0.1% to 0.01% of total plated cells. After enzymatic digestion, these cells remained attached and could be re-cultured in hAS for several weeks without signs of proliferation. Conversely, when re-cultured in FBS or pooled human cord blood serum, these rounded cells generated a new confluence of MSCs highly similar to those obtained from the primary culture in terms of proliferative, clonogenic, and differentiation potential. As these rounded cells also showed angiogenic and apparently partial cardiomyogenic potential, they were first named mesodermal progenitor cells. Later, we developed a selective culture method capable of isolating MPCs with a high grade of purity, pushing the yield to around 1% of total plated cells, applying hydrophobic non-gas-treated plastics as the culture surface (Trombi et al., 2009). The establishment of optimal culture conditions for the isolation of MPCs from hBM allowed for a more comprehensive characterization of these intriguing cells. In 2010, a specific MPC phenotype was defined to distinguish them from standard MSCs by flow cytometry, resulting in SSC^low^SSEA-4^neg^CD105^bright^MSCA-1^+^CD90^bright^, while MPCs were SSC^high^SSEA-4^+^CD105^dim^MSCA-1^neg^CD90^neg^. Interestingly, pluripotency-associated genes, such as *OCT-4* and *NANOG* expression, were detected exclusively in MPCs, and *nestin* was consistently expressed (Pacini et al., 2010). Further investigations on the integrin profile later revealed the expression of specific α and β chains on MPCs. In particular, these cells showed podosome-like structures sustained by integrins αL (CD11a), αM (CD11b), αX (CD11c), and β2 (CD18) that were absent on MSCs, extending their phenotype characterization and functionally sustaining an increased adhesion onto activated and non-activated endothelium (Pacini et al., 2013).

Succeeding and more rigorous studies and MPC differentiation capability demonstrated that their mesengenic commitment takes place through an SSA-4^+^CD105^int^CD90^bright^ early intermediate precursor, named *early* MSCs, activating a specific Wnt5/calmodulin signaling pathway (Fazzi et al., 2011). After culturing MPCs for a week in mesengenic culture conditions, usually identified as “passage one MSC” (P1-MSC) cultures, cells can be detached and further expanded (P2-MSCs), definitively acquiring a *classic* or *late* MSC phenotype and proliferation/differentiation features. Interestingly, P2-MSC culture was independent of the activation of the Wnt5/calmodulin signaling pathway and was not affected by calmidazolium chloride (CLMDZ), a potent and specific calmodulin inhibitor that can completely suppress the differentiation of primary MPC cultures into P1-MSCs. Moreover, CLMDZ did not affect the endothelial differentiation of MPCs, resulting in a highly specific inhibition of MPC differentiation into *early* MSCs only.

**FIGURE 1:**
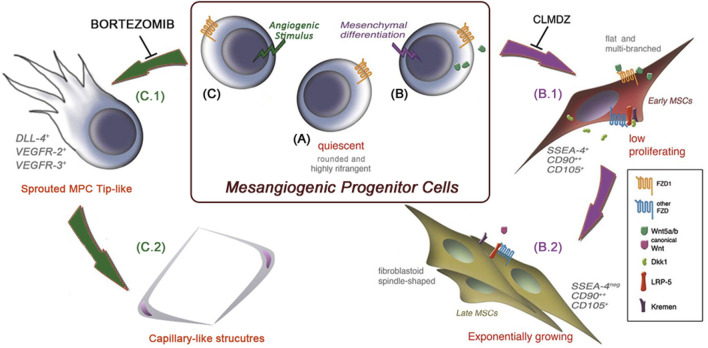
MPC mesengenic and angiogenic differentiation pathways are mutually exclusive. Mesangiogenic progenitor cells (MPCs) are isolated *in vitro* as quiescent adherent cells with a unique morphology resembling that of cultured macrophages **(A)**. Following the induction of mesenchymal differentiation **(B)**, MPCs rapidly activate the non-canonical Wnt signaling pathway and start to proliferate as flat and multi-branched cells **(B.1)**. As a result of canonical Wnt signaling activation, cells acquire the definitive MSC phenotype and functions **(B.2)**. Under angiogenic stimuli and independently of Wnt signaling activation **(C)**, MPCs proliferate and sprout from spheroids in 3D cultures, resembling the activity of endothelial tip cells **(C.1)**. Once sprouted, MPCs acquire the ability to form capillary-like tubes *in vitro* **(C.2)**. These two differentiation pathways are mutually exclusive, and shortly after mesengenic induction **(B)**, MPCs lose their angiogenic potential, and *vice versa*. (MSCs: mesenchymal stromal cells, CLMDZ: calmidazolium chloride).

Minimal criteria to characterize MPC cultures were definitively identified in 2016 (Montali et al., 2016a). Alongside the above-mentioned lack of MSC markers such as CD73 and specific integrin profile, phenotype should include CD31 and, surprisingly, CD45, from weak to brightly expressed in MPC. A percentage of CD73^neg^CD90^neg^CD45^+^CD31^+^of 95% has been indicated as the lower purity limit to consider the primary culture as an MPC culture. However, assaying the differentiation potential of these cultures represents a more stringent functional characterization. Together with the demonstration that MPCs can differentiate into P2-MSCs and are able to form calcium deposits in an osteogenic medium and intracellular lipid droplets in an adipogenic medium, MPCs should be capable of angiogenic sprouting from 3D-spheroids cultured in VEGF-rich medium. At this time, as the cardiomyogenic differentiation could not be definitively demonstrated, MPCs were then renamed as mesangiogenic progenitor cells, maintaining the same acronym. More recently, the MPC angiogenic potential and endothelial differentiation capability were clearly reported *in vivo*, applying MPC constructs on chicken chorioallantoic membrane (Montali et al., 2017). In this work, we also showed that MPCs can uptake acetylated low-density lipoproteins (Ac-LDL) and trans-endothelial migration but do not directly form capillary-like tubes (CLTs) in gels composed of extracellular matrix proteins. These CLTs were obtainable only if MPCs were first allowed to sprout from 3D-spheroids under VEGF stimulus in a 2-step differentiation protocol similar to the P1 and P2 steps of mesengenic differentiation. As a further similarity with the mesengenic differentiation pathway, bortezomib, a potent 26S proteasome inhibitor, has been demonstrated to specifically block MPC sprouting from spheroids but not subsequent CLT formation (Pacini et al., 2019). Interestingly, we also demonstrated that the mesengenic and vasculogenic potentials of MPCs are mutually exclusive, as P2-MSCs completely lost any angiogenic potential. Figure 1 summarizes both MPC differentiation pathways.

## 2 Pop#8, the *in vivo* progenitor of MPCs in human bone marrow

**FIGURE 2:**
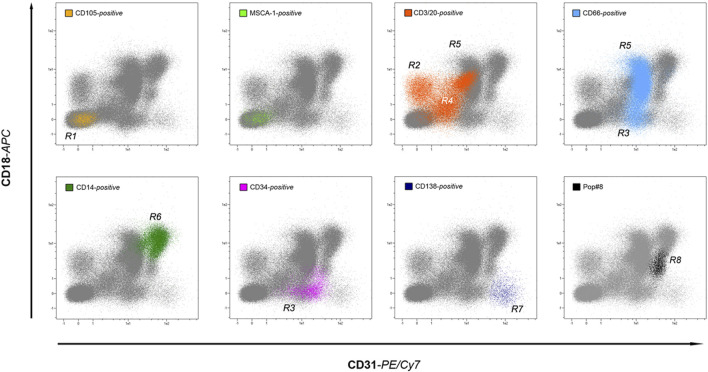
A roadmap to *in vivo* MPCs. By applying lineage-specific markers, the backgating method allows identifying most of the human bone marrow-resident leukocyte populations in the CD18 vs. CD31 dot-plot. Interestingly, the seven lineages investigated: *non-hematopoietic* (yellow and green dots), *lymphoid* (orange dots), *myeloid* (light blue dots), *monocytic* (green dots), *plasmacytoid* (dark blue dots), and *hematopoietic stem/progenitors* (purple dots) occupy distinct regions of the plot, without significant overlap. In addition, an eighth region is occupied by lineage-negative cells (black dots), generically identified as *population #8* (Pop#8). Sorting experiments have demonstrated that Pop#8 represents a unique cell fraction capable of generating MPCs in culture.

In 2015, our efforts were focused on clarifying whether MPCs arise from a unique *in vivo* progenitor and eventually identifying it in human adult BM aspirates (Pacini et al., 2016). Starting from the MPC phenotype, we first fractionated BM-MNCs into three different subpopulations: CD105^+^MSCA1^+^, CD105^+^MSCA-1^neg^, and CD31^+^MSCA-1^neg^ and cultured them for 6–7 days in pooled human AB-type serum (PhABS)-containing medium, resembling the MPC-selective conditions except for the application of hydrophilic TC-treated plastics, in order to create MPC-promoting and MSC-permissive culture conditions. As predicted by their immunophenotype *in vitro*, MPCs were detected only in the cultured CD31^+^MSCA-1^neg^ fraction but, interestingly, without any signs of fibroblastoid MSC-like cells despite the permissive attaching properties of the substrate. As expected, MSC-like cells were instead detected proliferating in the cultured CD105^+^MSCA-1^+^ fraction. However, in human adult BM, a plethora of mononuclear cells express CD31, including various precursors and mature elements of the myeloid lineage. Even if immunomagnetic cell sorting confirmed the existence of MPC-progenitor(s) in the CD31^+^CD14^+^/CD66^neg^, a more in-depth investigation of CD31-positive sub-fractions was needed. To this end, we used flow cytometry to analyze hBM-MNCs combining CD31 with another myeloid marker that was also positive on MPCs, such as CD18. Interestingly, as both CD31 and CD18 have been expressed by hBM-MNCs at three different levels of fluorescence intensity (*dim*, *intermediate,* and *bright*), plotting events in a CD31 vs. CD18 cytogram allowed visualization of a “map” of distribution of the various positive subpopulations. Then, a reverse gating strategy employing lineage markers identified seven regions of the plot associated with specific bone marrow populations, including lymphocytes (CD3/CD20), granulocytes (CD66), monocytes (CD14), plasma cells (CD138), and hemopoietic and endothelial stem/progenitor cells (CD34). Nonetheless, a further recognizable population CD31^bright^CD18^dim^ was found to be negative for any of the lineage markers applied (Figure 2). We then demonstrated that this eighth population (*Pop#8*) was the unique bone marrow fraction able to generate MPCs in culture with PhABS-containing medium. Characterization of *Pop#8* revealed a homogeneous population expressing high levels of CD31 and CD64, weak expression of CD33, CD13, and CD45, and negative for the lineage marker CD14.

## 3 Rise and fall of the MPCs from human bone marrow cultures

**FIGURE 3:**
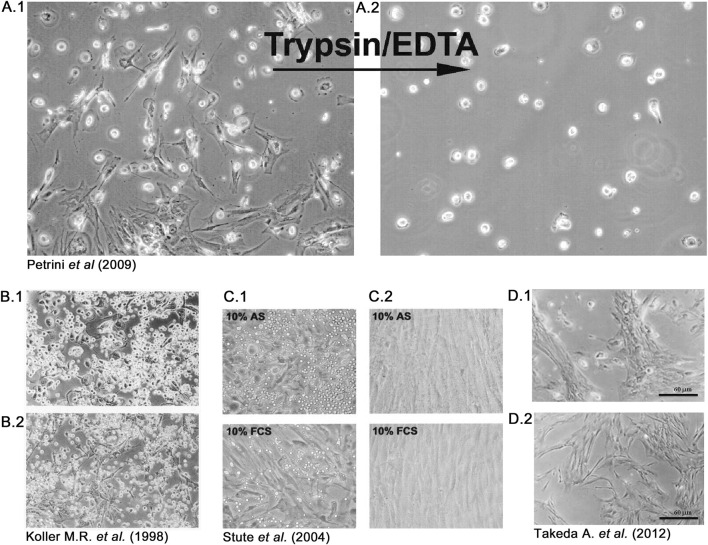
Rounded and trypsin-resistant cells were frequently observed in human bone marrow cultures when applying human serum. In 2009, our group described *mesangiogenic progenitor cells* (MPCs) as rounded and refringent cells co-isolated with *mesenchymal stromal cells* (MSCs), exclusively in human bone marrow cell cultures supplemented with human sera **(A.1)**. After trypsin digestion, MPCs remaining firmly attached to the primary culture flasks and were consequently lost during passaging **(A.2)**. Previously, Koller et al. recorded a similar observation using both human plasma **(B.1)** and serum **(B.2)**, and Stute et al. described monocyte-like “contaminating” cells only in autologous serum (AS) and not in fetal calf serum (FCS) supplemented cultures **(C.1)**, also recording that these rounded cells were lost during passaging **(C.2)**. Takeda et al. reported the latest description of MPC-like cells in 2012, comparing cultures supplemented with human serum **(D.1)** or FCS **(D.2)**.

At the end of the 20th century, the evidence of the multipotency of an easily expandable progenitor of the mesenchymal lineage with apparent “stem” properties, the mesenchymal stromal/stem cells (MSCs) (Pittenger et al., 1999; Bruder et al., 1994), attracted great interest from the scientific community and triggered a decade of great efforts translating MSCs “from the bench to the bedside” (Bunpetch et al., 2017). In parallel to the increasing evidence of the great therapeutic value of *in vitro* expanded MSCs, serious concerns regarding the safety of cell manufacturing procedures have been expressed. In particular, the use of FBS has been indicated as one of the principal possible sources of morbidity in MSC-based cell therapies (Spees et al., 2004; Heiskanen et al., 2007), together with elevated batch-to-batch variability, high price, and ethical issues (Subbiahanadar Chelladurai et al., 2021). Culture media substitutes first considered were human-derived autologous and allogeneic sera (HS), and we explored this possibility when we recorded the first observations regarding the culture of MPCs. However, that first observation of “contaminating” rounded and highly refringent cells in hBM-MNCs cultured with HS (Figure 3A.1), not detected in FBS-supplemented cultures, was not a singular observation. Indeed, some authors reported similar results when applying human plasma or serum. In 1998, Koller et al. noted that applying human plasma- (Figure 3B.1) or serum-supplemented media (Figure 3B.2) did not produce a confluence of stromal cells but instead showed numerous large and adherent cells not observed in animal serum-containing medium that resembled the MPC morphology (Manfredr et al., 1998). Later, Stute et al. reported that from 0.5% to 4.0% of “monocytes” and other, non-specified, rounded cells were able to be co-isolated together with classical spindle-shaped mesenchymal cells in primary cultures of hBM-MNCs (Figure 3C.1) (Stute et al., 2004), showing very similar characteristics to our first identification and characterization of MPCs (Petrini et al., 2009) (Figure 3A.2). The authors reported that passaging produced a drastic reduction of these “contaminating” cells, as they remained firmly adhered to the surface of the culture plate after trypsin digestion of the primary cultures (Figure 3C.2). A few years after, Takeda et al. also reported a “non uniform morphology” of adherent cells in the primary culture of human bone marrow-derived cells applying autologous HS, in comparison to FBS-containing medium (Takeda et al., 2012). Photomicrographs from this latest paper clearly show MPC-like cells in the HS-containing cultures (Figure 3D.1) but not in FBS-supplemented cultures (Figure 3D.2). When not more specifically described, these trypsin-resistant rounded cells were generically identified as hemopoietic in origin and considered undesired contaminating cells that were lost during passaging of MSC cultures (Stute et al., 2004; Codinach et al., 2016).

As most of these efforts were intended to expand MSCs, these parallel observations did not attract sufficient attention, even from the scientists who recorded them. Our breakthrough interpretation of the aforementioned experimental results describing MPCs, reported in the last 15 years, suggests that these previously “unwanted” cells could retain significant biological value and become of great interest in the field of MSC-based clinical applications and tissue regeneration if there were characterized as MPCs (Pacini, 2014). Moreover, it has also been hypothesized that the presence of MPCs could be responsible for the controversial data regarding the mesengenic and angiogenic potential of MSCs cultured in HS, where different culture conditions could select or simply promote the persistence of co-isolated MPCs as well as their direct progenies (Pacini and Petrini, 2014). Notwithstanding, the descriptions of co-isolated cells with different morphologies and phenotypes in human bone marrow MSC cultures rapidly decreased when alternative culture supplementation to whole serum was investigated.

The large-scale production of MSCs in HS has significant limitations affecting manufacturing, regulatory issues, and quality assurance. Even though a considerable number of studies have demonstrated the feasibility of using HS when culturing MSCs, the great biological variability of the produced HS batches has led to contradictory results (Sotiropoulou et al., 2006; Dimarakis and Levicar, 2006; Berger et al., 2006). Applying pooled HS seems not to significantly reduce this variability generated by donor-related intrinsic (sex, age, and blood groups) and extrinsic (diet and lifestyle habits) factors, as well as by different manufacturing processes (off-the-clot vs. plasma-derived, cryopreservation, etc.).

Since 2006, promising results have been obtained by first applying platelet-rich plasma (PRP) and then adding thrombin (tPRP) to maximize the release of growth factors from the platelet granules. However, the use of media supplementation with PRP or tPRP also has some drawbacks, such as the incomplete release of granules content and mainly the presence of fibrinogen, which leads to possible clot activation. These aspects could be ameliorated by applying human platelet lysate (hPL) (Schallmoser et al., 2007), the production and application of which could be standardized (Shih and Burnouf, 2015). Moreover, it has been demonstrated that hBM-MSCs can be expanded with increased population doublings and growth rate in hPL-supplemented cultures compared with FBS, HS, or tPRP (Bieback et al., 2009; Kinzebach et al., 2013). Consequently, even with some issues to face, hPL has been widely considered the most feasible substitute for FBS in MSC GMP-grade expansion for clinical applications (Altaie et al., 2016). This has led to a progressive reduction in the number of studies applying HS in culturing bone marrow-derived MSCs and, accordingly, relegating our identification of the MPCs and their multipotency to a single observation, which did not attract sufficient attention from experts in the field, who were mainly focused on the promising MSC clinical applications. However, the interesting properties shown by MPCs led us to investigate the neglected application of HS more deeply, shifting the focus to these cells. As described above, we especially applied commercially available pooled human AB-type serum (PhABS) in order to isolate purified MPCs, representing an upstream approach in culturing bone marrow-derived cells, usually intended for amplifying MSCs instead (Trombi et al., 2009). However, even though applying selective culture conditions has allowed obtaining at least 90% of MPCs, differences in isolation performance have been noted from different PhABS batches. Subsequently, we recorded a satisfactory reproducibility in MPC isolation by imposing more stringent criteria for the selection of commercially available PhABS. These selection criteria are still valid and include the following: i) declaration of the country of donor origin (partially reducing variability related to donor diet and other lifestyle habits); ii) male-only donors, preventing demonstrated exposure sources uniquely relevant to female donors (Phillips et al., 2024); and iii) an off-the-clot manufacturing process.

In 2016, by screening some commercially available PhABS, we demonstrated that differences in the content of a number of growth factors (hGF) could be the basis of the variability in the isolating performance reported (Montali et al., 2016b). In particular, we showed that applying PhABS batches with elevated concentrations of EGF, FGF-2, VEGF-A, and PDGF-AB resulted in a higher percentage of MSCs co-isolated under MPC-selective culture conditions, worsening the MPC isolating performance. Today, the pre-culture screening of PhABS batches allows isolating MPCs with purity ranging from 95% to 99%. Moreover, studying the expression of hGF receptors on isolated MPCs and MSCs, we also hypothesized that some hGFs could promote MSC proliferation while others could trigger MPC differentiation. The growing applications of hPL in hBM-MSC primary cultures have led to a widespread consensus in applying culture conditions particularly rich in the aforementioned hGF (Fonseca et al., 2021; Oeller et al., 2021). However, under those culture conditions, MPCs could rapidly differentiate toward highly proliferating MSCs and, in the end, vanish during the very first days of culture. This has contributed to the idea that those rounded and firmly attached cells should be considered “contaminating” cells of hemopoietic origin emerging in particular during isolation when applying human sera.

Even though it is strongly believed by the author that MPCs should not be considered “undesired contaminating cells” but rather increasing the regenerative potential of the BM cell preparations (Giannotti et al., 2013), their hemopoietic origin should not be excluded. MPC morphology evokes that of cultured macrophages (Mϕ) or dendritic cells (DCs) and some of the expressed markers (i.e., CD31). Functions (Ac-LDL uptake, trans-endothelial migration, and ECM degradation) reported in order to demonstrate the early angiogenic potential of MPCs are also shared by these latest cells and overlapping endothelial progenitor cell (EPC) traits, as well (Rohde et al., 2005; Zhang et al., 2006). In a recent paper, Barachini et al. highlighted some peculiar features ascribable only to BM-derived MPCs and not to macrophages and DCs adhered to the culture flask in culture from adipose tissue or cord blood. In particular, the resulting MPCs were CD14-negative, consistently expressed *nestin,* and showed dispersed podosomes (Barachini et al., 2021). These latest peculiarities also distinguish MPCs from other monocyte-derived Mϕ-like adherent cells that were previously described as retaining a mesengenic differentiation potential. Interestingly, these latest cells share the expression of *osteopontin* (SPP1) and *matrix metalloproteinase-9* (MMP-9) (Zhang and Shively, 2010; Fadini et al., 2012).

Thus, the retention of mesangiogenic potential in the hemopoietic cell compartment, particularly within the monocyte/macrophage subsets, is surprising but not completely unprecedented. This hypothesis has been supported by interesting but underestimated data reported by several groups, mainly during the first decade of the 2000s, which will be discussed next. In particular, there is neglected evidence showing that circulating monocytes, conventionally believed to differentiate only into macrophages and dendritic cells, can differentiate into a variety of non-phagocytic cells, supporting the idea of a broader plasticity of this blood cell subset.

## 4 Plasticity of the *circulating* monocyte/macrophage compartment

Circulating monocytes are highly adaptable cells that give rise to other cell types, which differ in phenotype and function, and they are, for that reason, considered a progenitor cell type. They originate from hemopoiesis via the common myeloid progenitor (CMP) and the granulocyte/monocyte progenitor (GMP), which represent the precursor populations for monoblasts. Monoblasts are immature monocytes, and their offspring migrate from the bone marrow into the peripheral blood. Circulating monocytes can differentiate into several types of macrophages once they have infiltrated into tissues (Gordon and Taylor, 2005). Under specific stimuli, monocytes can differentiate into macrophages, acquiring an inflammatory phenotype and producing cytokines, radicals with antimicrobial activity, and proteinases while exerting elevated phagocytic activity. Under different stimuli, macrophages undergo a functional switch to an anti-inflammatory and regeneration-promoting phenotype supporting wound healing processes by stimulation of angiogenesis, matrix production, and cell proliferation (Novak and Koh, 2013). It has also been reported that macrophages associated with certain carcinomas adopt a further alternative phenotype capable of promoting tumor progression (Stout et al., 2009; Ricketts et al., 2021). Notably, early developmental studies in the 1990s demonstrated the emergence of macrophages during embryonic development before the establishment of definitive hemopoiesis, suggesting that certain tissue-resident macrophages are independent of monocyte invasion and are able of self-renewing (Mizoguchi et al., 1992; Yamanada et al., 1991; Sorokin and Hoyt, 1992). Consequently, a new interpretation of these data suggests that monocytes infiltrating inflamed tissues could not only support the expansion of the macrophage and dendritic cell population but possibly exert functions that cannot be performed by resident macrophages or DC and acquire very distinct functional profiles (Guilliams et al., 2018).

The mechanisms underlying this macrophage functional heterogeneity are still a matter of debate. It could be the consequence of monocyte differentiation toward different pathways, or alternatively, macrophages are plastic cells able to exert different activities in response to specific micro-environmental conditions (functional plasticity hypothesis). In any case, it is reasonable to hypothesize that the regenerative potential of the monocyte/macrophage compartment could be much greater than previously thought. As mentioned above, approximately during the first decade of the 2000s, several groups proposed specific culture conditions for differentiating human circulating monocytes into non-phagocytic Mϕ-like cells that demonstrate multipotent differentiating capability. In the following sub-paragraphs, the most relevant multipotent monocyte/macrophage-derived cell populations are described in comparison with MPCs.

### 4.1 Monocyte-derived multipotential cells (MOMCs)

In 2003, Kuwana M. et al. reported the isolation of a primitive cell population named *monocyte-derived multipotential cells* (MOMCs), from circulating CD14-positive cells in humans. MOMCs have been characterized, in culture, by the expression of CD14, CD68, CD45, and CD34 but also type I/III collagen, showing a fibroblast-like morphology and mesenchymal differentiation capability (Kuwana et al., 2003). MOMCs are isolated from peripheral blood mononuclear cells (PB-MNCs) cultured in DMEM/10% FBS on fibronectin-coated plastics, and their origin from circulating CD14-positive cells has been confirmed by fluorescent cell tracking and cell depletion. Interestingly, MOMCs retain the ability to differentiate into phagocytes. A consistent angiogenic potential, both *in vitro* and *in vivo*, has also been reported (Kuwana et al., 2006). MOMCs share several peculiar features with MPCs in terms of Ac-LDL uptake, retention of the mesengenic/angiogenic potential, and very similar phenotype characterized by weak expression of CD14, CD45, CD13, and CD105 and consistent expression of CD31, CD11b, CD11c, and HLA-I/II. Their morphology looks very similar in light and electronic microscopy, and both these cell populations express pluripotency-associated genes such as *OCT-4* and *NANOG* (Seta and Kuwana, 2010). However, differences between MOMCs and MPCs are evident. First, these two cell populations are generated from different *ex vivo* progenitors: circulating CD14-positive cells for MOMCs and BM-derived CD14-negative cells for MPCs. Second, MPCs are CD34-negative and require human serum supplementation; conversely, CD34-positive MOMCs are cultured with FBS on fibronectin-coated plastics. The *ex vivo* progenitors of MOMCs have not been fully identified as different circulating precursors. They could be included in the CD14-positive fraction, although an alternative mechanism leading to these cells could be hypothesized. Indeed, Seta N. et al. did not exclude that different and specific culture conditions could drive circulating monocytes toward a “de-differentiation” pathway, acquiring a more immature phenotype and differentiation properties.

### 4.2 Programmable cells of monocytic origin (PCMOs)

The idea that peripheral blood monocytes could acquire plasticity following an *in vitro* de-differentiation has been successively supported by the report of *programmable cells of monocytic origin* (PCMOs). These cells are obtained from circulating monocytes isolated by plastic adherence from peripheral mononuclear cells in RPMI-1640 supplemented with 10% human-type AB serum. The subsequent treatment with low doses of M-CSF and IL-3 for 6 days led to proliferating PCMOs capable of differentiation into neohepatocytes and pancreatic islet-like cells (Ruhnke et al., 2005). Moreover, PCMOs express some endothelial features (Hutchinson et al., 2007), and recent data suggest that these cells are also able to differentiate *in vitro* toward osteoblast-like cells (Açil et al., 2017) and collagen type-II-producing chondrocytes (Pufe et al., 2008). Interestingly, similar to MPCs and MOMCs, PCMOs express *OCT-4* and *NANOG*, but the de-differentiation process has been analyzed in more detail at the molecular level. Ungefroren et al. demonstrated that the reactivation of *OCT-4* and *NANOG* genes in peripheral monocytes is essential to generate PCMOs, combined with the expression of other reprogramming factors like *KLF4* and *MYC*, but not *SOX2* (Ungefroren et al., 2010; Ungefroren et al., 2015a). This molecular signature is identical to what is reported in MPCs (Pacini et al., 2010). Moreover, Ungefroren H et al. also showed that after “reprogramming” in a PCMO-inducing medium, the percentage of CD14-positive cells varies from 90%-95% to around 45%, ranging from 30% to 60%, while other markers like CD86 and HLA-DR remained constant over the culture time, suggesting the emergence of CD14^neg^CD86^+^HLA-DR^+^ sub-population precisely matching the MPC phenotype. These data strongly suggest that the induction of PCMOs from circulating monocytes is exerted by the acquisition of the MPC-specific phenotype and their peculiar and immature molecular signatures.

### 4.3 Human circulating fibrocytes

In the early 90s, by applying a murine wound healing model, Bucala et al. identified and isolated a blood-borne fibroblast-like cell type named *fibrocytes* (Bucala et al., 1994). Similarly to MOMCs, fibrocytes are isolated from peripheral blood mononuclear cells by culture in DMEM supplemented with 20% FBS on fibronectin-coated plastics, and their mesengenic potential has been demonstrated *in vitro* and *in vivo* (Hong et al., 2005; Hong et al., 2007). The phenotype of these cells shows a great overlap with MOMCs and PCMO phenotypes. Fibrocytes bear features of both fibroblasts and monocytes expressing CD34, CD45, and CD11, as well as type I and type III collagen. Moreover, it has been demonstrated that fibrocytes could be generated from CD14-positive enriched fraction of PB-MNCs (Abe et al., 2001), specifically from the CD11b^+^CD115^+^Gr1^+^ sub-population of monocytes (Niedermeier et al., 2009). Compared to MOMCs and PCMOs, fibrocytes attracted more interest, perhaps due to their earliest identification, and these cells have been shown to play a role in different tissue fibrosis both in experimental and clinical settings (Herzog and Bucala, 2010). The bone marrow origin of fibrocytes has been definitively demonstrated by sex-mismatched or GFP-tagged bone marrow transplantation in mice. In these studies, after reconstitution of male bone marrow in female hosts or engraftment of GFP-tagged transplant, the isolated fibrocytes from PB-MNCs and wounded tissue showed positive hybridization to the Y-chromosomes specific probe or GFP fluorescence emission, respectively (Quan et al., 2004; Mori et al., 2005). However, the frequency of circulating cells showing a fibrocyte-related phenotype is very low, from 0.1% to 0.5% of white blood cells (Metz, 2003). Optimized culture conditions could lead to one-log fold higher culture yields (Pilling et al., 2009). Even if not yet rigorously discussed due to the lack of sorting experiments, the previously reported possibility of obtaining fibrocytes from CD14-positive circulating monocytes seems to support the idea of “differentiation” from circulating monocytes as the origin of *ex vivo* fibrocytes. However, the phenotypic and functional features of these cells, such as decreasing CD14 expression, induction of CD34, and acquisition of multilineage differentiation potential, support the hypothesis of “de-differentiation,” similar to what has been proposed for MOMCs and PCMOs.

### 4.4 Monoosteophils

In 2010, Zhang and Shively described a further bone-forming cell population isolated *in vitro* from the circulating CD14-positive fraction and called them *monoosteophils* (Zhang and Shively, 2010). Data showed that culturing human circulating monocytes in RPMI-1640 supplemented with 10% FBS and LL-37, the active product of the proteolysis of the cathelicidin hCAP-18, forms large adherent macrophage-like cells distinct from M1, M2-polarized macrophages, and dendritic cells. Monoosteophils show close similarities with an MPC-like phenotype characterized by the weak expression of CD14 and CD45, the absence of both CD34 and CD90, both expressing CD49c (Integrin α3) and producing high levels of *osteopontin* (SPP1) and *matrix metallopeptidase 7* and 9 (MMP-7, MMP-9) (Pacini et al., 2010; Zhang and Shively, 2013; Zhang et al., 2020). Monoosteophils are capable of bone formation both *in vitro* and *in vivo*, as well as accelerating the process of bone repair in murine models. Due to the limited amount of data regarding monoosteophil differentiation, genuine mesengenic potential has not been demonstrated. However, the epiphyseal-like structure reported in ectopic monoosteophils-HA/TCP implants suggests that these cells could retain a wider mesengenic potential. Monoosteophils have been discussed as distinct from MOMCs, fibrocytes, or MCCs. The latter is described in the next paragraph; however, this statement is based only on the different expression levels of CD14 and CD34. Once again, the generation of monoosteophils has been proposed as a monocyte differentiation pathway alternative to the M1, M2 polarization or DC and osteoclast maturation. Nonetheless, the acquisition of the multiple differentiating potentials toward non-phagocytic cell lineages as a consequence of a “de-differentiation” process should not be excluded, as the pluripotency-associated molecular signature has not yet been investigated.

### 4.5 Myeloid calcifying cells (MCCs)

In 2005, Eghbali-Fatourechi et al. described 1% to 2% of circulating osteoblast-like cells in PB-MNCs of adults and even higher percentages in adolescents. These cells were detected and sorted for the expression of *osteocalcin* (OC) and *bone alkaline phosphatase* (BAP), demonstrating an osteogenic potential both *in vitro* and *in vivo* (Eghbali-Fatourechi et al., 2005). Similarly to MOMCs and fibrocytes, circulating OC^+^BAP^+^ cells can be cultured in 15% FBS-containing media on fibronectin-coated plastics, but at the time of this first observation, their origin was unclear. Later on, Fadini et al. showed that OC^+^BAP^+^ cells originate from the myeloid lineage and express monocyte/macrophage markers such as CD45, CD14, and CD68; thus, the authors renamed these cells as *myeloid calcifying cells* (MCCs) (Fadini et al., 2011). MCCs are around 10-fold enriched in bone marrow compared to peripheral blood. Moreover, in sex-mismatched bone marrow transplantation, it has been demonstrated that these cells are long-lived and resistant to myeloablation. Due to the lack of CD34, MCCs have been described as distinct from fibrocytes and MOMCs, while the greatest similarities were reported with respect to the CD34-negative monoosteophils, which also express OC but not BAP.

### 4.6 Circulating CD14^+^CD34^low^

During the same year, by applying cell sorting techniques, Romagnani et al. demonstrated that the CD14^+^CD34^low^ peripheral blood leukocyte sub-population represents the majority of the circulating KDR^+^ cells as well as the main source of circulating endothelial progenitor cells (cEPCs) (Romagnani et al., 2005). The authors also demonstrated, *in vitro*, the clonogenicity and multipotency of these cells, showing osteogenic, adipogenic, and neurogenic differentiating potential, and concluding that circulating CD14^+^CD34^low^ cells exhibit the phenotypes and functional features of stem cells. This conclusion has also been supported by the revealed consistent expression of *NANOG* and *OCT-4*. Interestingly, the authors described circulating CD14^+^CD34^low^ as distinct from MOMCs, considering that these cells could also be isolated from peripheral mononuclear cells after depletion of CD34^+^ once cultured on fibronectin. However, even if not discussed in this paper, the “de-differentiation hypothesis” could explain a possible relationship between CD14^+^CD34^low^ and MOMCs, where the latter could also be generated from CD14^+^CD34^neg^ undergoing de-differentiation and starting to express CD34. Coupling angiogenic and mesengenic potential, particularly osteogenesis, among the circulating CD34-positive fraction has also been demonstrated in 2006 by Matsumoto et al. (2006). In this article, the authors did not analyze CD14 expression and demonstrated the mesangiogenic potential of the CD34-positive fraction *in toto*. However, flow cytometric analysis demonstrated that more than 95% of sorted cells expressed CD31 and also CD45 at a weaker florescence intensity, as well as *Pop#8*, but lacked KDR positive stain. Once again, the authors did not account for the “de-differentiation hypothesis” and suggested that this circulating CD34-positive cell plasticity could arise from different cell differentiation pathways of a heterogeneous population, trans-differentiation, or even cell fusion mechanisms.

Taken together, all this underestimated data strongly supports the idea that the circulating monocyte/macrophage cell subset retains, *in vitro*, an angiogenic/vasculogenic potential coupled with mesengenic potential. However, the many different culture conditions and supplementation, as well as cell selections (summarized in Table 1), lead to the acquisition of this plasticity, contributing to the great confusion and consequent decline of interest in the field. It is the author’s opinion that most of this confusion originates from the general way in which most researchers discussed their findings. Focusing on the differences that distinguish all of the cell identities described above, highlighting peculiarities, and naming them in many alternative ways (fibrocytes, MOMCs, MCCs, monoosteophils, PCMOs, etc.) makes it difficult not to consider any of these reports as single and peculiar observation. However, all these multipotent cell populations of monocyte/macrophage origins show a few relevant similarities (Table 1) that could be more deeply discussed to shed further light on the possible mechanisms sustaining this unconventional plasticity. In general, the loss of expression of some lineage markers like CD14 and CD45 in favor of the acquisition of more immature ones, that is, CD34 and CD90, represents a common finding. The expression of pluripotency-associated genes *OCT-4* and *NANOG* has been reported even for MOMCs and PCMOs (Seta and Kuwana, 2010; Açil et al., 2017; Pufe et al., 2008). As already mentioned, this strongly suggests that the acquisition of plasticity by a circulating monocytes/macrophages subset could be sustained by “trans-differentiation” processes made possible through the “de-differentiation” stage induced by particular culture conditions.

**TABLE 1:** Most relevant multipotent monocyte/macrophage-derived cell populations compared to MPCs. Differences in isolation methods and culture conditions have led to the definition of distinct cell identities. However, some important similarities should be discussed. In particular, the reduced expression of lineage markers CD14 and CD45 in favor of the acquisition of pluripotency-associated gene expression seems to be a common finding.

	MPCs	MOMCs	PCMOs	Fibrocytes	Monoosteophils	MCCs	CD14^+^CD34^low^
**Cell source**	Bone marrow mononuclear cells	Peripheral CD14-positive cells	Peripheral mononuclear cells	Peripheral CD14-positive cells	Peripheral CD14-positive cells	Peripheral mononuclear cells	Peripheral mononuclear cells
***Ex vivo* progenitor phenotype**	CD34^neg^CD45^low^CD14^neg^	CD14^+^	?	CD14^+^CD11b^+^CD115^+^Gr1^+^	CD14^+^	CD34^neg^CD45^+^CD14^+^	CD14^+^CD34^low^
**Serum supplementation**	Human serum	Fetal bovine serum	Human serum	Fetal bovine serum	Fetal bovine serum	Fetal bovine serum	Fetal bovine serum
**Cytokines and other supplements**	-	-	M-CSF and IL-3	-	LL-37	β-GlyPhosph, Dexa, AA	VEGF
**Culture surface treatment**	No gas-treated plastics	Fibronectin coating	Gas-treated plastics	Fibronectin coating	Not reported	Fibronectin coating	Fibronectin coating
**Pluripotency-associate gene expression**	OCT-4, NANOG, KLF4, MYC	OCT-4, NANOG	OCT-4, NANOG, KLF4, MYC	Not investigated	Not investigated	Not investigated	OCT-4 , NANOG
**CD45 expression**	Weak	Positive	Positive	Positive	Weak	Not investigated	Weak
**CD14 expression**	Weak	Weak	Mostly negative	Weak	Weak	Positive	Positive
**CD34 expression**	Negative	Positive	Positive	Positive	Negative	Negative	Weak
**CD90 expression**	Negative	Negative	Mostly positive	Not investigated	Negative	Not investigated	Not investigated
**Multilineage differentiation potential**	Osteo-, adipo-, chondro-, and vasculogenic	Osteo-, adipo-, chondro-, neuro-, and vasculogenic	Hepato-, osteogenic, and pancreatic islet-like cells	Not investigated	Osteogenic	Osteogenic	Osteo-, Adipo-, Neuro-, and Vascuologenic

M-CSF: macrophage colony-stimulating factor; IL-3: interleukin 3; β-GlyPhosph: β-glycerophosphate; Dexa: dexamethasone; AA: ascorbic acid; VEGF: vascular endothelial growth factor.

**FIGURE 4:**
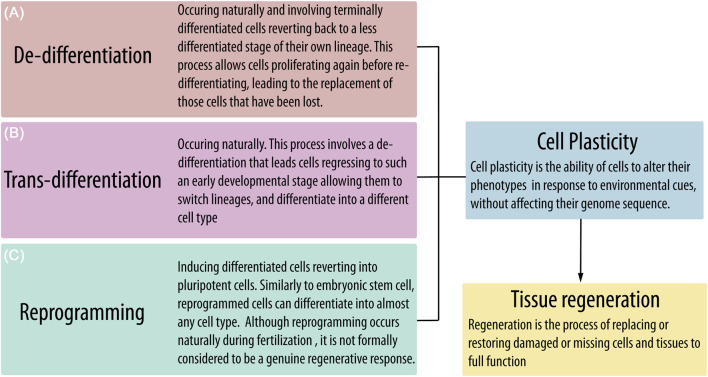
Tissue homeostasis and function could be impaired by senescence and injuries, leading to the loss of important resident cell populations. The potential to restore tissue function by replacing damaged or missing cells is related to the presence and activation of plastic progenitors that can proliferate and differentiate in response to environmental cues. However, mature cells could also acquire cell plasticity through naturally occurring processes such as **(A)** de-differentiation and **(B)** trans-differentiation or by **(C)** reprogramming after forced expression of some pluripotency-associated genes.

De-differentiation has been described as the process by which terminally or partially differentiated cells revert to a less differentiated stage of their own lineage. *In vivo*, this allows cells to proliferate again and replace lost cells through re-differentiation. De-differentiation may occur naturally in response to tissue damage, sustaining regeneration also in mammals (Jopling et al., 2011). However, the de-differentiation process could also lead mature cells to such an early developmental stage, allowing them to switch lineage and differentiate into another cell type. This latest process has been indicated as “trans-differentiation” and also occurs naturally in response to injuries of different tissues like the liver, pancreas, and heart (Merrell and Stanger, 2016). *In vitro*, the best known and most widely applied example of de-differentiation and trans-differentiation is represented by “reprogramming,” in which forced expression of a few transcription factors, including *OCT-4* and *NANOG*, can revert terminally differentiated cells into pluripotent cells (Jopling et al., 2011) (Figure 4). However, both de-differentiation and trans-differentiation induced *in vitro*, not by altering the expression of Yamanaka factors, have been reported as a consequence of specific culture conditions for a number of adult cells, such as osteocytes (Sawa et al., 2019), chondrocytes (Ghosh et al., 2022), adipocytes (Liu et al., 2021; Radhakrishnan et al., 2019), hepatocytes (Yang et al., 2002), and pancreatic cells (Hunter and Stein, 2017). Thus, it is reasonable to hypothesize that similar processes could be implemented in circulating monocytes/macrophages by the specific culture conditions cited above, enabling reactivation of *OCT-4* and *NANOG* expression. This hypothesis has already been suggested for PCMOs, although the authors stated that de-differentiation in response to M-CSF/IL-3/human serum exposure could not reflect a physiological mechanism (Ungefroren et al., 2015b). This intriguing hypothesis could be extended to MOMC and C14^+^CD34^low^ circulating cells, in which reactivation of *OCT-4* and *NANOG* has been demonstrated, but possibly also to other cell identities discussed here and in which pluripotency-associated markers have not yet been investigated.

## 5 Can MPCs and their *in vivo* ancestor be considered physiologically prone to trans-differentiation?

Considering the hypothesis that the circulating monocyte/macrophage compartment could acquire plasticity by *in vitro* de-differentiation, the many peculiar features shared by mesangiogenic progenitor cells (MPCs), PCMOs, MOMCs, and monoosteophils suggest that MPCs could represent the cell identity toward which the de-differentiation process reprograms the circulating mature phagocytic compartment. Even if this hypothesis should be corroborated by comparing the cell molecular signature, the constitutive expression of *OCT-4* and *NANOG* but not *SOX2* in MPCs suggests that the characteristic “adult *Oct-4* circuits,” described as the possible molecular mechanism sustaining the MPC plasticity (Pacini et al., 2010), could be activated during the de-differentiation process. The activation of the peculiar transcriptional complex *Oct-4/Sox15* and the homodimer *Oct-4/Oct-4*, described in the “adult *Oct-4* circuits,” induces consistent expression of their target genes, such as *FBX15*, *SPP1* (*osteopontin*), and *nestin,* considered among the most specific markers of the MPC identity. Thus, it would be of great interest to evaluate the expression of these latest markers and the “adult *OCT-4* circuit” activation in de-differentiated circulating monocytes/macrophages, possibly clarifying a common molecular mechanism. Currently, this hypothesis is rather speculative because, in the few plastic cell identities where *OCT-4* expression has been reported (MOMCs, PCMOs), none of the possible target genes was tested. Conversely, in monoosteophils where consistent expression of *SPP1* was reported, pluripotency-associated markers were not assayed.

However, MPCs can be isolated exclusively from human bone marrow (Barachini et al., 2021) by applying selective culture conditions (Trombi et al., 2009) that do not involve the use of any exogenous stimulating factors like M-CSF/IL-3 for PCMOs, LL-37 for monoosteophils, or fibronectin coating for MOMCs and MMCs in addition to human serum supplementation. This evidence, together with the very short culture time needed to isolate non-proliferating MPCs, also suggests that these cells do not undergo a process of de-differentiation during the *in vitro* selection, but instead, their plasticity could represent an intrinsic capacity already retained by their *in vivo* ancestors. The latter is supported by evidence of comparable expression of *OCT-4* and *NANOG* in *Pop#8*, which is described as the only bone marrow cell population able to generate MPCs *in vitro*. Consequently, *Pop#8* could represent a bone marrow resident in an immature stage of monocytic lineage physiologically prone to trans-differentiation.

This novel assumption could have relevant implications for the definition of the differentiation plasticity of the hemopoietic compartments, which represents a field of knowledge still widely debated after more than two decades. However, experiments designed to verify the possible trans-differentiation capability of the hemopoietic cells usually involved clonogenic hemopoietic stem cells (HSCs) in order to demonstrate different lineages of clonal reconstitution after transplantation in irradiated mice (Graf, 2002). Nonetheless, according to the hypothesis discussed here, the trans-differentiation of the hemopoietic bone marrow compartment could also be exerted by non-clonogenic progenitors/precursors like monoblasts, which the *Pop#8* cells closely resemble (Pacini et al., 2016).

## 6 Concluding remarks

MPCs are promising cells in the field of musculoskeletal tissue regeneration, not only for their mesengenic differentiation capability but also for their vasculogenic potential. Indeed, regenerating tissues that are poorly vascularized in origin, like cartilage and tendon, or after injuries, such as in non-consolidating bone, is still a difficult challenge to face in cell therapy. In recent years, due to their ease of expansion, MSCs have attracted almost entirely the attention of experts in the field. However, the clinical application of MSCs has been disappointing, and we are still waiting for an effective therapy based on these cells (Česnik and Švajger, 2024). Indeed, studies that reproducibly and reliably demonstrate the effectiveness of MSCs in clinical settings are still lacking.

The primary factor contributing to this data inconsistency is the significant heterogeneity of MSC preparations (Chen et al., 2024). This heterogeneity can arise from inherent biological differences among tissue sources and donors (Levy et al., 2020), as well as from biological variations in growth media supplement batches and discrepancies in local manufacturing processes (Shaz et al., 2024). Furthermore, the extended culture time required to expand MSCs exacerbates the effects of these variations in culture determinants on the final cell product. After many efforts to establish clinical-grade expansion of MSCs, the growing practice of applying alternatives to human serum supplementation may have considerably decreased the number of observations of MPC-like cells in bone marrow cultures.

However, the author strongly believes that relegating MPCs to mere contaminants and underestimating the plasticity of monocytes and their progenitors could be a mistake. A renewed interest in isolating MPCs, testing the reproducibility of culture methods, identifying their clonal identity in human bone marrow, and rigorously tracking their plasticity could contribute to identifying MPCs as a new player in the cell-based regeneration of musculoskeletal tissues.

After more than two decades of expanding MSCs to obtain a significant number of cells to transplant, a paradigm shift may be needed. Even if the clinical value of MPCs should be rigorously demonstrated in pre-clinical and clinical studies, the discovery of these cells suggests a novel approach where “less could be more.” Fewer cells means reduced *in vitro* manipulation and risk of transformation, possibly preserving the plasticity of multipotent cells that naturally reside in the tissue of origin.

## Data Availability

The original contributions presented in the study are included in the article/supplementary material; further inquiries can be directed to the corresponding author.
